# Supplementary material for: A Machine Learning Model Based on First-Trimester Lipidomic Signatures for Predicting Metabolic Pregnancy Complications
Source: Int J Mol Sci. 2025 Dec 7;26(24):11824. doi: 10.3390/ijms262411824 (PMC12733181; doi:10.3390/ijms262411824)
Supplement: Supplementary file 1 [file ijms-26-11824-s001.zip › SuplFigures.docx]

(a)
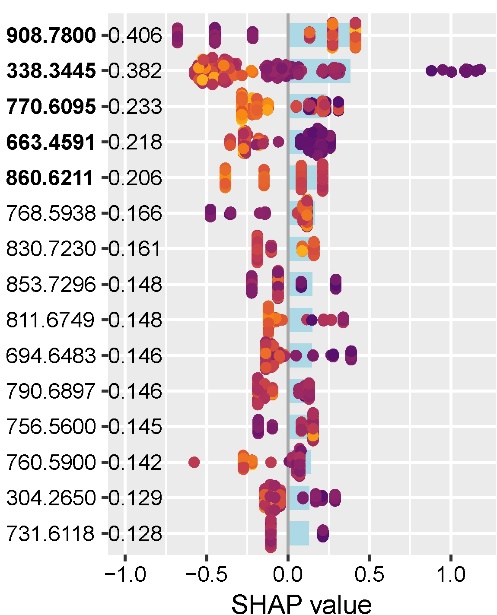
(b)
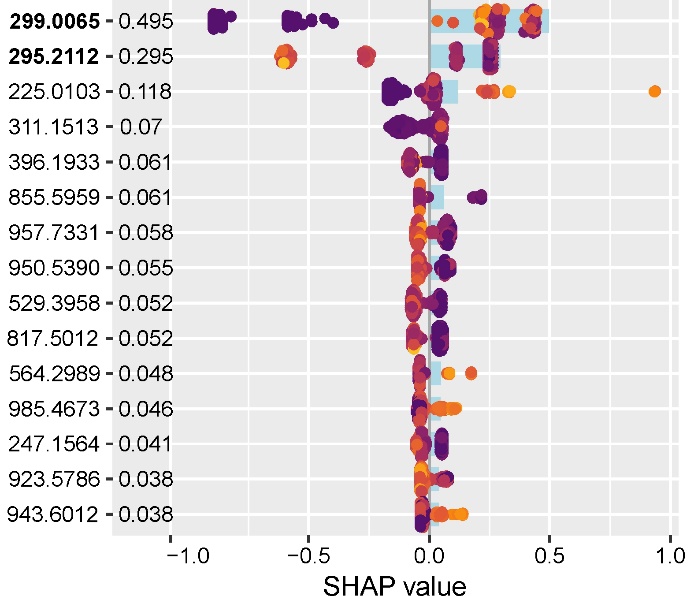


(c)
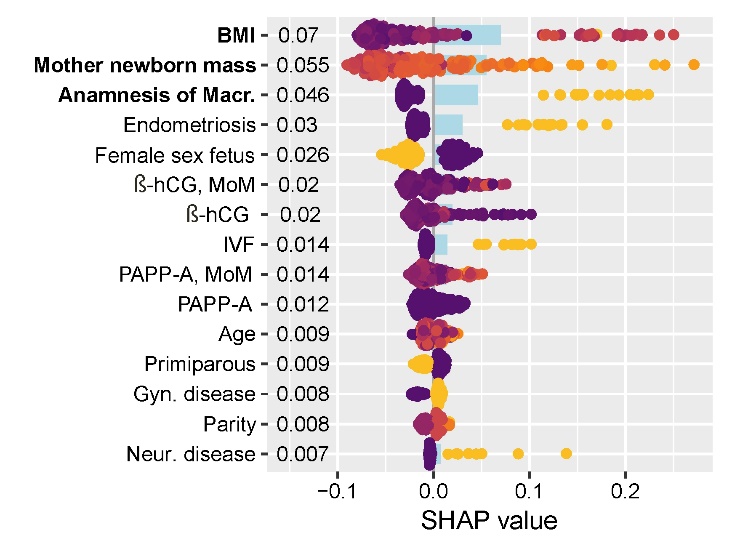
(d)
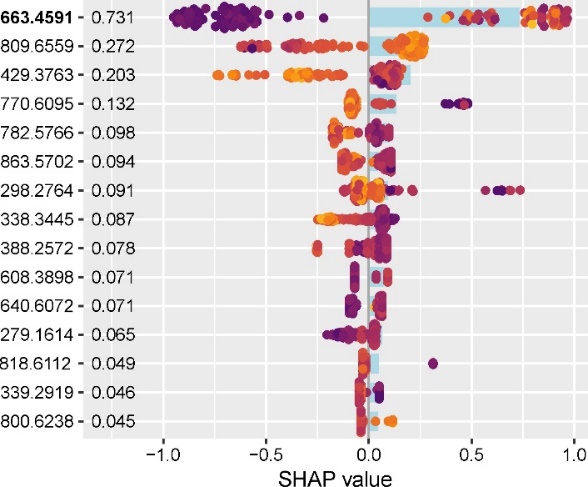


(e)
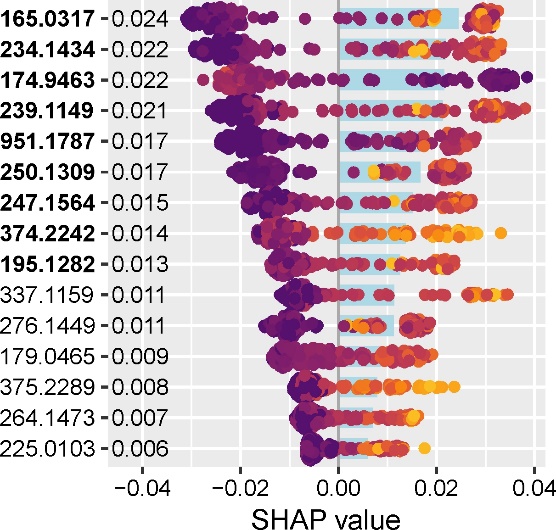
(f
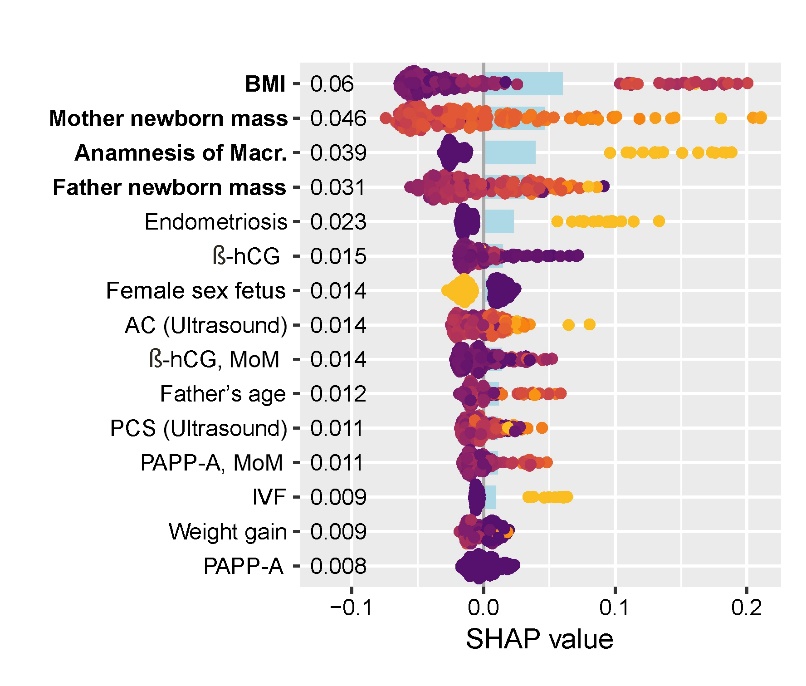


**Figure S1.** The 15 features with the highest contribution to the model, based on the mean absolute Shapley value. Features identified as potential markers with mean of SHAP value across samples no less than half of maximum SHAP value in the model are highlighted in bold. Yellow color is associated with the highest values of feature (intensity in case of m/z peaks), purple color is associated with the lowest values of feature (intensity in case of m/z peaks). Features from mass-spectrometry lipidomic spectra are labeled by their m/z value in the spectra, features from clinical data are labeled by their names. (a) XGBoost, GDM, positive ion mode; (b) XGBoost, GDM, negative ion mode; (c) Random Forest, GDM, clinical data; (d) XGBoost, macrosomia, positive ion mode; (e) Random Forest, macrosomia, negative ion mode; (f) Random Forest, macrosomia, clinical data.


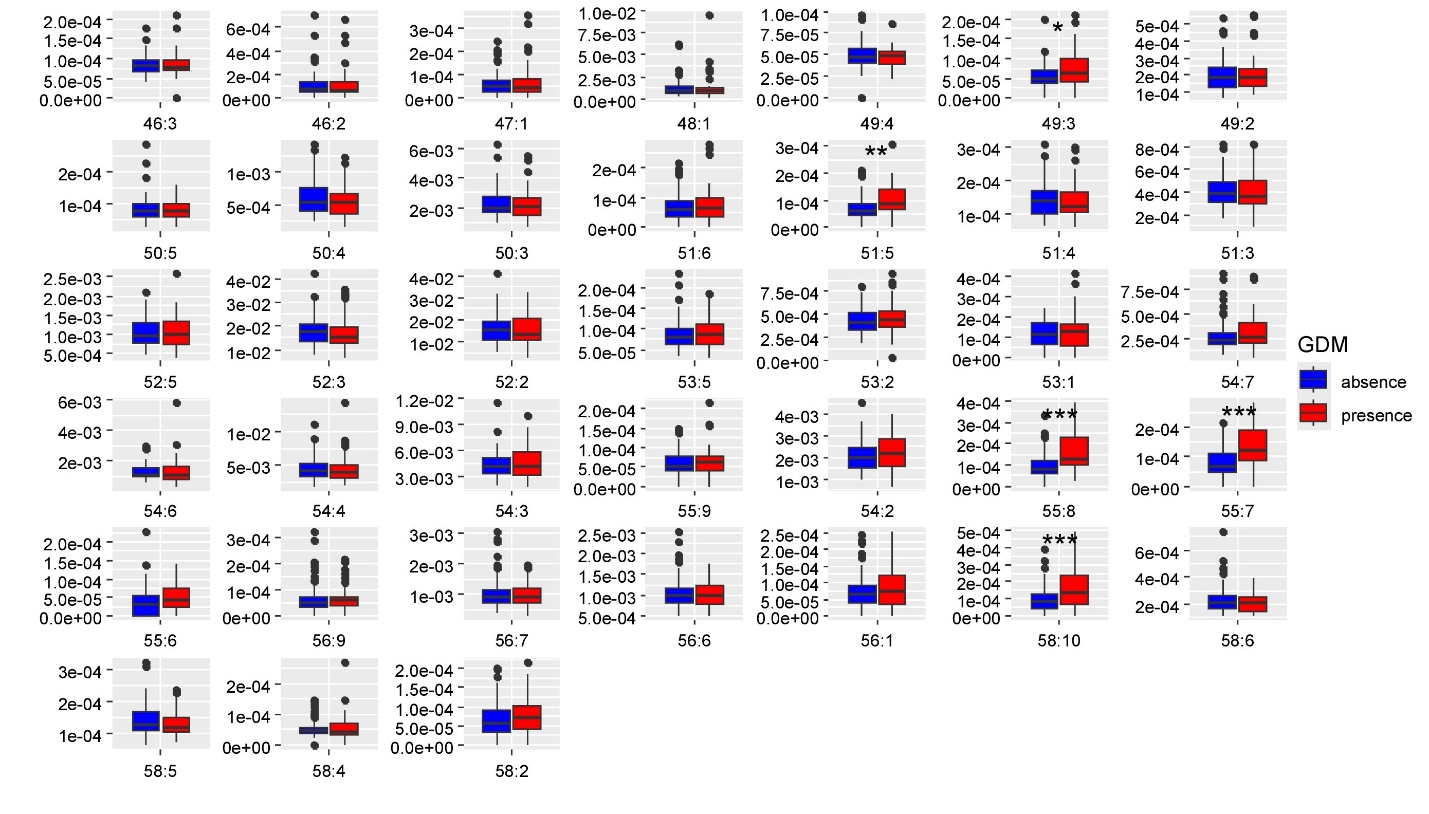


**Figure S2.** Levels of triacylglycerols in case of absence of GDM and presence of GDM. *** - p <0.001, * - p <0.05. P is calculated by Manna-Whitney test.


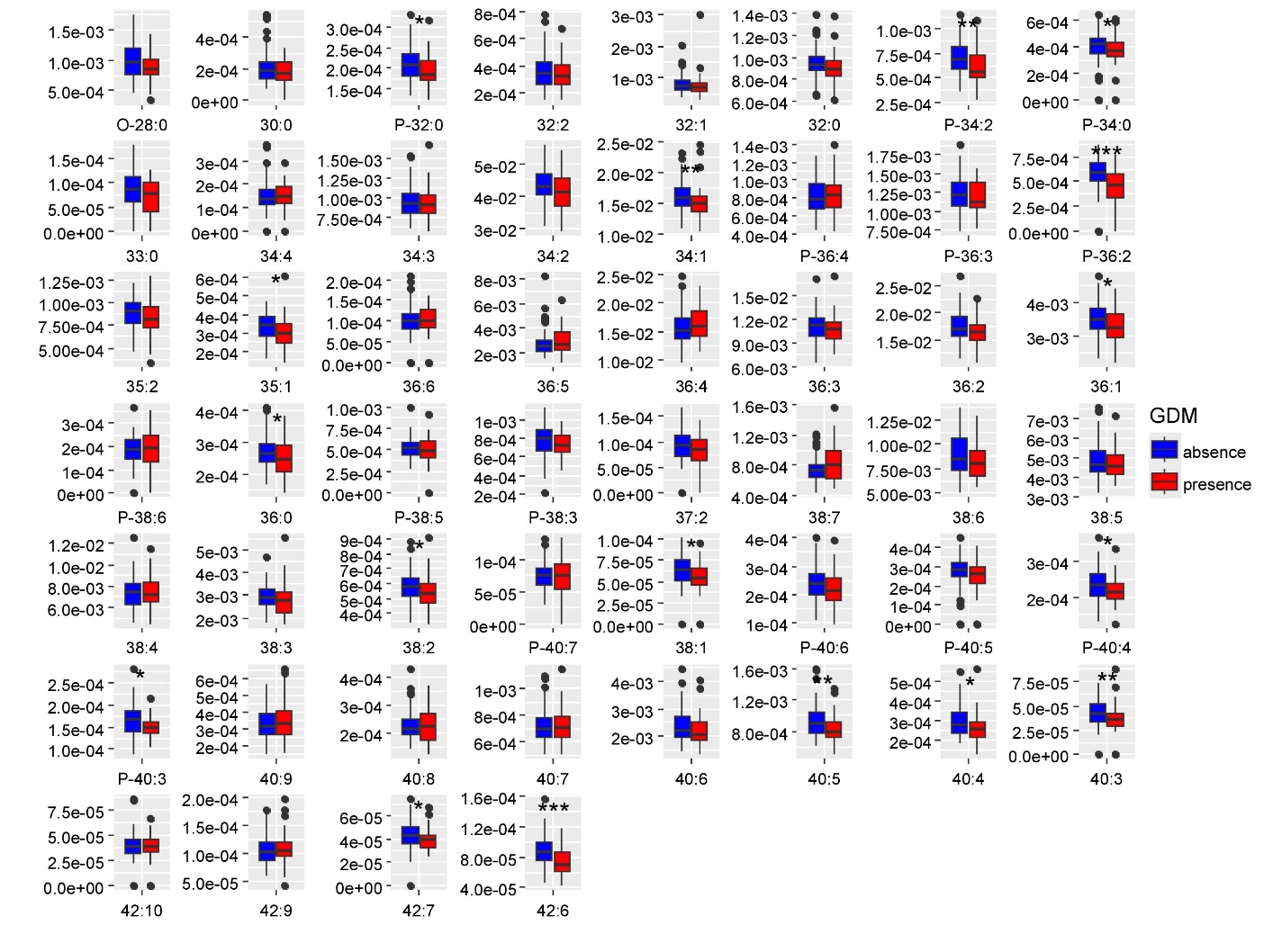


**Figure S3.** Levels of phosphatidylcholines in case of absence of GDM and presence of GDM. ** - p < 0.01, * - p <0.05. P is calculated by Manna-Whitney test.


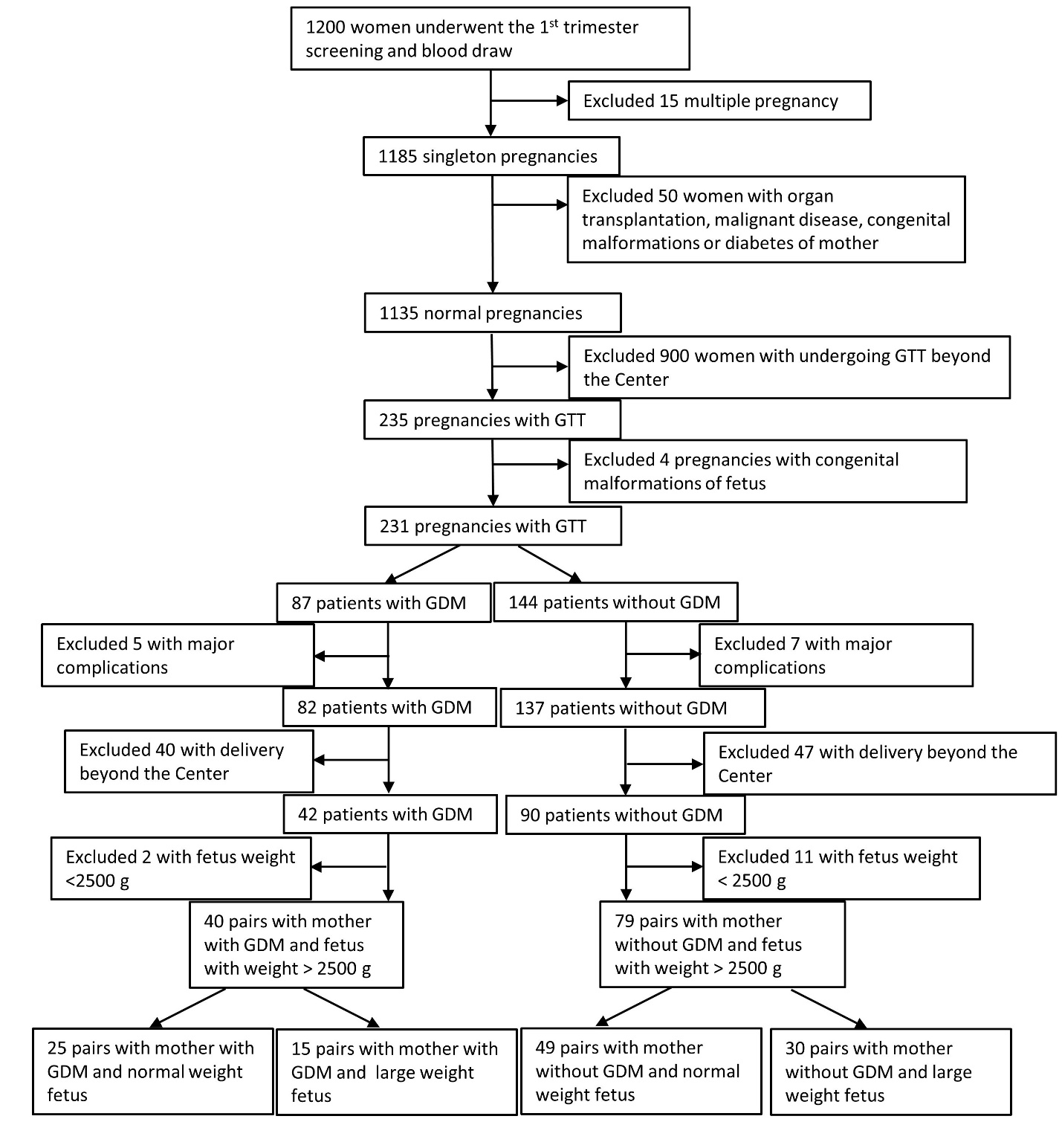


**Figure S4.** Consort flow diagram of patients monitoring, exclusion from analysis and dividing in four groups. GDM is gestational diabetes mellitus, GTT is glucose tolerance test, Center is the V.I. Kulakov National Medical Research Center for Obstetrics, Gynecology, and Perinatology.
