# Supplementary material for: A Machine Learning Model Based on First-Trimester Lipidomic Signatures for Predicting Metabolic Pregnancy Complications
Source: Int J Mol Sci. 2025 Dec 7;26(24):11824. doi: 10.3390/ijms262411824 (PMC12733181; doi:10.3390/ijms262411824)
Supplement: Supplementary file 1 [file ijms-26-11824-s001.zip › Table S1.docx]

Table S1. Clinical characteristics of women in each study group. Numerical parameters are pre-sented as Med (Q1, Q3), categorical parameters are presented as "absolute number" ("percentage within group"%). P – p-value for the comparison of multiple samples using the Kruskal-Wallis test for numerical parameters and Pearson's chi-square test for categorical parameters. Pi-j – statistical significance for pairwise comparison using Dunn's test for numerical parameters and pairwise chi-square test. Parameters that were statistically significant in the multiple sample comparison are highlighted in bold.

| **Clinical Parameter** | **GDM -, M - (Group 1, n = 49)** | **GDM +, M - (Group 2, n = 25)** | **GDM -, M + (Group 3, n = 30)** | **GDM +, M + (Group 4, n = 15)** | **P-value** |
| --- | --- | --- | --- | --- | --- |
| Medical History | | | | | |
| IVF, n (%) | 0 (0%) | 3 (12%) | 5 (17%) | 3 (20%) | 0.03 |
| Birth weight (mother), kg | 3.34 (3.15; 3.5) | 3.5 (3.15; 3.65) | 3.7 (3.5; 4.1) | 3.6 (3.5; 3.92) | 0.001  p_1-3_=0.001  p_1-4_=0.02 |
| Maternal BMI before pregnancy, kg/m² | 20.8 (19.4; 21.8) | 22.2 (20.1; 24.0) | 22.6 (19.8; 25.5) | 27.0 (23.7; 29.0) | <0.001  p_1-3_=0.03  p_1-4_<0.001  p_2-4_=0.02 |
| Father's age, years | 33 (30; 35) | 36 (30; 38) | 34.5 (31.3; 38.8) | 35 (32; 41.5) | 0.04 |
| Birth weight (father), kg | 3.5 (3.3; 3.7) | 3.57 (3.4; 3.9) | 3.8 (3.52; 4) | 3.8 (3.5; 4.1) | 0.03  p_1-3_=0.03 |
| DM in relatives, n (%) | 12 (24%) | 9 (36%) | 8 (27%) | 6 (40%) | 0.57 |
| Cardiovascular diseases, n (%) | 7 (14%) | 6 (24%) | 3 (10%) | 1 (7%) | 0.38 |
| Vascular diseases, n (%) | 5 (10%) | 2 (8%) | 2 (7%) | 2 (13%) | 0.89 |
| Blood diseases, n (%) | 6 (12%) | 1 (4%) | 1 (3%) | 0 (0%) | 0.23 |
| GI diseases, n (%) | 10 (20%) | 8 (32%) | 7 (23%) | 4 (27%) | 0.74 |
| Kidney diseases, n (%) | 11 (22%) | 6 (24%) | 5 (17%) | 3 (20%) | 0.91 |
| Endocrine diseases, n (%) | 8 (16%) | 5 (20%) | 2 (7%) | 6 (40%) | 0.05 |
| GDM in history, n (%) | 1 (2%) | 2 (8%) | 0 (0%) | 3 (20%) | 0.02 |
| Autoimmune diseases, n (%) | 1 (2%) | 2 (8%) | 0 (0%) | 0 (0%) | 0.23 |
| Neurological diseases, n (%) | 2 (4%) | 2 (8%) | 3 (10%) | 2 (13%) | 0.61 |
| Regular menstrual cycle, n (%) | 44 (90%) | 22 (88%) | 26 (87%) | 13 (87%) | 0.97 |
| Gynecological diseases, n (%) | 33 (67%) | 19 (76%) | 26 (87%) | 12 (80%) | 0.27 |
| Polycystic ovary syndrome, n (%) | 1 (2%) | 1 (4%) | 3 (10%) | 1 (7%) | 0.46 |
| Polyps, n (%) | 0 (0%) | 3 (12%) | 4 (13%) | 1 (7%) | 0.08 |
| Endometrial hyperplasia, n (%) | 0 (0%) | 1 (4%) | 0 (0%) | 0 (0%) | 0.28 |
| Benign ovarian tumors, n (%) | 7 (14%) | 3 (12%) | 5 (17%) | 2 (13%) | 0.97 |
| Endometriosis, n (%) | 2 (4%) | 2 (8%) | 7 (23%) | 4 (27%) | 0.02 |
| Chronic endometritis, n (%) | 3 (6%) | 5 (20%) | 3 (10%) | 4 (27%) | 0.11 |
| Parity, n (%) | 0 - 22 (45%)  1 - 23 (47%)  2 - 3 (6%)  3 - 1 (2%) | 0 - 12 (48%)  1 - 10 (40%)  2 - 3 (12%)  3 - 0 (0%) | 0 - 10 (33%)  1 - 17 (57%)  2 - 3 (10%)  3 - 0 (0%) | 0 - 4 (27%)  1 - 6 (40%)  2 - 4 (27%)  3 - 1 (7%) | 0.32 |
| Gravidity | 2 (1; 3) | 2 (1; 4) | 2 (2; 3) | 2 (1.5; 3) | 0.91 |
| Number of deliveries | 2 (1; 2) | 2 (1; 2) | 2 (1; 2) | 2 (1; 3) | 0.32 |
| Nulliparous, n (%) | 21 (43%) | 12 (48%) | 9 (30%) | 4 (27%) | 0.37 |
| Primigravida, n (%) | 12 (24%) | 8 (32%) | 6 (20%) | 4 (27%) | 0.78 |
| Macrosomia in history, n (%) | 1 (2%) | 1 (4%) | 8 (27%) | 6 (40%) | <0.001  p_1-3_=0.02  p_1-4_=0.002 |
| First Trimester | | | | | |
| Maternal age, years | 30 (28; 33) | 32 (29; 35) | 32 (28.25; 35) | 32 (28; 34.5) | 0.38 |
| ß-hCG, IU/l | 51.3 (38.3; 64.4) | 46.9 (28.5; 73.1) | 52.5 (27.33; 67.92) | 44.7 (25.9; 79) | 0.94 |
| ß-hCG, MoM | 1.39 (0.94; 1.88) | 1.35 (0.86; 2.21) | 1.57 (0.96; 2.06) | 1.41 (0.91; 2.5) | 0.90 |
| PAPP-A, IU/l | 3.62 (1.91; 5) | 2.82 (2.29; 4.08) | 3.35 (2.56; 4.43) | 1.85 (1.1; 3.58) | 0.13 |
| PAPP-A, MoM | 1.27 (0.81; 1.7) | 1.12 (0.94; 1.58) | 1.44 (0.98; 1.71) | 0.98 (0.71; 1.74) | 0.51 |
| Weight gain by 1st trimester, kg | 1 (0; 2) | 0 (0; 1) | 0 (0; 1.45) | 0.6 (0; 1) | 0.19 |
| CRL, mm | 61 (57; 65.9) | 62 (58; 65.5) | 62.65 (55; 66.88) | 61 (57.5; 63) | 0.81 |
| BPD, mm | 20 (19; 22.2) | 21 (20; 21.7) | 20.75 (19.12; 22.72) | 21 (19.85; 21) | 0.95 |
| HC, mm | 74 (70; 82) | 74.3 (71; 79.6) | 73.55 (70.25; 82.75) | 71 (70; 78) | 0.82 |
| AC, mm | 59 (56; 66) | 60 (57.2; 64) | 62.45 (56.25; 67.77) | 63 (58; 64.05) | 0.73 |
| NT, mm | 1.5 (1.3; 1.6) | 1.5 (1.5; 1.6) | 1.5 (1.33; 1.6) | 1.6 (1.45; 1.7) | 0.41 |
| Placental thickness, mm | 13 (12; 15) | 13 (12; 14) | 13 (12; 13.75) | 14 (14; 14.5) | 0.18 |
| Female fetal sex, n (%) | 25 (51%) | 14 (56%) | 11 (33%) | 4 (27%) | 0.19 |
| Delivery Outcomes | | | | | |
| Gestational age at delivery, weeks | 39.3 (38.4; 40.2) | 38.6 (38.3; 39.5) | 39.55 (38.7; 40.08) | 38.5 (38.15; 39.75) | 0.06 |
| Cesarean section, n (%) | 10 (20%) | 15 (60%) | 19 (63%) | 8 (53%) | <0.001  p_1-2_=0.001 p_1-3_=0.001 |
| Emergency CS, n (%) | 4 (8%) | 5 (20%) | 9 (30%) | 1 (7%) | 0.05 |
| Neonatal birth weight, g | 3324 (3170; 3430) | 3210 (3010; 3370) | 4084 (4026.25; 4223) | 4020 (3810; 4145) | <0.001  p_1-3_<0.001  p_1-4_<0.001  p_2-3_<0.001  p_2-4_<0.001 |
| Apgar score - 1 minute | 8 (8; 8) | 8 (8; 8) | 8 (8; 8) | 8 (8; 8) | 0.67 |
| Apgar score - 5 minutes | 9 (9; 9) | 9 (9; 9) | 9 (9; 9) | 9 (9; 9) | 0.39 |
| Hospital stay (newborn), days | 3 (2; 4) | 4 (3; 5) | 4 (3; 5) | 3 (3; 4) | <0.001  p_1-2_=0.002  p_1-3_<0.001 |
| Hospital stay (mother), days | 3 (3; 4) | 4 (4; 5) | 4 (4; 5) | 4 (3; 5) | <0.001  p_1-2_=0.002 p_1-3_<0.001 |
